# tDCS over the left inferior frontal cortex improves speech production in aphasia

**DOI:** 10.3389/fnhum.2013.00539

**Published:** 2013-09-06

**Authors:** Paola Marangolo, Valentina Fiori, Maria A. Calpagnano, Serena Campana, Carmelina Razzano, Carlo Caltagirone, Andrea Marini

**Affiliations:** ^1^Facoltà di Medicina, Università Politecnica MarcheAncona, Italy; ^2^Department of Clinical and Behavioural Neurology, Istituto di Ricovero a Carattere Scientifico Fondazione Santa LuciaRoma, Italy; ^3^Department of Neurology, Universitá di Tor VergataRoma, Italy; ^4^Dipartimento di Scienze Umane, Università di UdineUdine, Italy

**Keywords:** tDCS, speech production, aphasia recovery, stroke, language rehabilitation

## Abstract

In this study, we investigated the combined effect of transcranial direct current stimulation (tDCS) and an intensive Conversational therapy treatment on discourse skills in 12 persons with chronic aphasia. Six short video clips depicting everyday life contexts were prepared. Three videoclips were used to elicit spontaneous conversation during treatment. The remaining three were presented only before and after the therapy. Participants were prompted to talk about the contents of each videoclip while stimulated with tDCS (20 min 1 mA) over the left hemisphere in three conditions: anodic tDCS over the Broca's area, anodic tDCS over the Wernicke's area, and a sham condition. Each experimental condition was performed for 10 consecutive daily sessions with 14 days of intersession interval. After stimulation over Broca's area, the participants produced more Content Units, verbs and sentences than in the remaining two conditions. Importantly, this improvement was still detectable 1 month after the end of treatment and its effects were generalized also to the three videoclips that had been administered at the beginning and at the end of the therapy sessions. In conclusion, anodic tDCS applied over the left Broca's area together with an intensive “Conversational Therapy” treatment improves informative speech in persons with chronic aphasia. We believe that positive tDCS effects may be further extended to other language domains, such as the recovery of speech production.

## Introduction

Failure to spontaneously produce fluent and informative speech is the most persistent disabling consequence after stroke, particularly in persons with aphasia with left anterior hemispheric lesions (SPREAD, 2012). Traditional linguistic-based therapies have proved reasonably effective (Jensen, 2000; Kemmerer and Tranel, 2000; Raymer and Ellsworth, 2002; Wambaugh et al., 2002; Marangolo, 2012). However, in many cases a severe reduction of the ability to produce informative speech does persist (Basso, 2010; Marangolo, 2010; Andreetta et al., 2012). For this reason, several efforts have been devoted to the development of new approaches aimed at enhancing the use of language in daily-life communicative situations (e.g., Ulatowska et al., 1983; Saffran et al., 1989; Glosser and Deser, 1990; Nicholas and Brookshire, 1993). Among these, “Conversational therapy” is probably one of the most used (Holland, 1991; Lai, 1993; Basso, 2010; Marini and Carlomagno, 2004; Vigorelli, 2007; Marangolo, 2010; Wilkinson and Wielaert, 2012). Within the conversational therapy approach the therapist and the person with aphasia are engaged in a natural conversation and the latter is encouraged to use all of his/her communicative means to convey informative speech (Grice, 1975; Basso, 2010; Marangolo, 2010).

Parallel to this growing interest in the way language is processed in daily communicative interactions, the traditional views of how to assess language deficits in persons with aphasia have been challenged. Several studies have shown that traditional standardized aphasia tests may not be sensitive enough to adequately assess linguistic deficits and recovery patterns in persons with aphasia (Larfeuil and Le Dorze, 1997). As a result, both functional and structural methods for the analysis of connected language samples from people with aphasia have been proposed (see Armstrong, 2000; Prins and Bastiaanse, 2004; Marini et al., 2011). One procedure for quantifying information content was originally developed by Yorkston and Beukelman (1980). They administered the Cookie Theft Picture description task (Goodglass and Kaplan, 1972) to a group of participants with aphasia. The levels of informativeness of these language samples were quantified in terms of Content Units (C-Units), clusters of elements and/or isolated phrases not always accompanied by a verb, but with high communicative value (Loban, 1966).

Over the last few years, converging evidence has suggested the usefulness of therapies associating intensive language treatment with brain stimulation. Indeed, persons with aphasia exhibit greater recovery of lexical-retrieval deficits when the language treatment is coupled with repeated transcranial magnetic stimulation (rTMS; Naeser et al., 2005, 2010, 2011; Martin et al., 2009; Cotelli et al., 2011) or transcranial direct current stimulation (tDCS; Baker et al., 2010; Fiori et al., 2011; Fridriksson et al., 2011; Kang et al., 2011; Marangolo et al., 2013; see Elsner et al., 2013 and Monti et al., 2013 for reviews). However, these studies did not demonstrate whether the improvements found in the naming tasks would enhance the individuals' ability to use language in daily life interactions (see Brady et al., 2012 for a review).

To the best of our knowledge, only four studies have reported spontaneous speech production in individuals receiving rTMS stimulation (Naeser et al., 2005; Martin et al., 2009; Barwood et al., 2011; Medina et al., 2012). However, even in these investigations the TMS was not coupled with concomitant language training and discourse productivity was merely quantified in terms of phrase length (Naeser et al., 2005; Martin et al., 2009; Barwood et al., 2011) and production of narrative words (Medina et al., 2012).

Considering the benefical effects of tDCS on lexical recovery (Monti et al., 2008; Baker et al., 2010; Fiori et al., 2011; Fridriksson et al., 2011; Marangolo et al., 2013), we hypothesize that a Conversational therapy coupled with repeated stimulation might induce significant linguistic improvements also on other aspects of language processing. Recent evidence suggests a potential role for Broca's area and the adjacent cortex in the processes of lexical selection and unification, that is the combination of word information into larger units that span multi-word utterances (e.g., Hagoort, 2005; Indefrey and Cutler, 2005; Marini and Urgesi, 2012). As such, the left Inferior Frontal Gyrus (LIFG) might play a pivotal role in the recovery of units with a high communicative value (i.e., Content Units). Then, it might be an ideal candidate for the stimulation during conversational therapy.

The present study was aimed to investigate linguistic and functional aspects of language recovery in 12 chronic participants with non-fluent aphasia whose linguistic production showed reduced information content and poor syntactic organization. Three different stimulation conditions were employed: the target condition included anodic stimulation of the Broca's area (i.e., LIFG); a control condition with anodic stimulation of the Wernicke's area (i.e., posterior portiong of the left superior temporal gyrus, LSTG) allowed us to control for the specificity of the effects obtained within the target condition; a further control condition included sham stimulation. We hypothesized that if the Broca's area is indeed involved in the recovery of informative words, we would find a greater improvement only in this condition. The linguistic skills were assessed using different approaches, namely standard aphasia testing and the analysis of speech samples obtained through the administration of a series of videoclips reproducing common everyday situations. In order to assess the extent of any potential recovery, the videoclips were also administered to a group of healthy individuals. This allowed us to further control for significant improvements in the group of aphasic participants with respect to normality.

Overall, this study aimed to determine the efficacy of tDCS coupled with Conversational therapy in improving the informative skills of the aphasic group and their ability to produce adequate content in terms of production of C-Units. We also hypothesized that the potential lexical improvement would be particularly evident for verbs, a category of content words that are particularly impaired in these patients and that are thought to play a crucial role in the structural formulation of sentences (see also Wambaugh et al., 2002).

## Materials and methods

### Participants

#### Control group

Twenty healthy individuals (10 males and 10 females) matched for age (40–75 years) and education level (13–17 years) with the aphasic group were enrolled in the experiment. All of them were native Italian speakers with no history of neurological or psychiatric illness.

#### Aphasic group

Twelve participants (8 males and 4 female) who had sustained a single left hemisphere stroke were included in the study. Inclusion criteria were native Italian proficiency, pre-morbid right handedness, a single left hemispheric stroke at least 6 months prior to the investigation, and no acute or chronic neurological symptoms requiring medication. The data analyzed in the current study were collected in accordance with the Helsinky Declaration and the Institutional Review Board of the IRCCS Fondazione Santa Lucia, Rome, Italy. Prior to participation, all participants signed informed consent forms.

### Neuropsychological assesment

The aphasic disorders were assessed using standardized language testing [the Battery for the analysis of aphasic disorders, BADA test (Miceli et al., 1994)] and the Token test (De Renzi and Vignolo, 1962). Participants were also administered different tasks to investigate the principal attentional functions [selective, divided and sustained attention tests (Zimmermann and Fimm, 1994)] and a visual memory test [the Ray Figure test (Orsini et al., 1987)] to exclude the presence of attention and memory deficits that might have biased their performance.

### Clinical data

**Figure 1:**
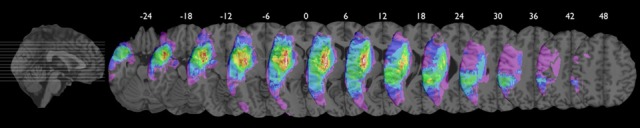
**Axial views of color coded probability map of lesion overlap (range 1% purple to 91% white).** Individual volume lesions were drawn manually on the re-oriented brain volume transformed into MNI standardized stereotaxic coordinate system using a computational semi-automatic procedure of REGISTER software provided by Brain Imaging Center, Montreal Neurological Institute, McGill University. Averaging the labeled voxels of the individual lesion volumes re-aligned in MNI space generated the probability map revealing the localization of areas of percentage of lesion overlap. Maximal overlap includes the capsula estrema, the claustrum, part of the capsula esterna and the putamen. The inferior frontal gyrus (including the Broca's area) and the superior temporal gyrus (including the Wernicke's area) were similary damaged both having about 45% of lesion overlap.

All participants had an ischemic lesion involving the left hemisphere. The lesion mapping analysis indicated that the areas of maximal lesion overlap were localized in the capsula estrema, the claustrum, part of the capsula esterna and the putamen (see Figure 1). The 12 participants were diagnosed with non-fluent aphasia as they had reduced verbal output in spontaneous speech. Their utterances were short and characterized by omissions of verbs and function words as well as errors in verb inflection. Patients with severe articulatory impairments were excluded, in order to avoid a possible confound in data analysis. Their basic comprehension skills were preserved and indeed they were able to engage in verbal exchanges with the therapist. All patients had some difficulties in word reading and writing and in comprehending complex verbal materials (Token test). In the noun and verb naming tasks, severe word-finding difficulties were present (see Table 1).

**Table 1:** **Sociodemographic and Clinical data of the 12 non-fluent aphasic participants**.

**Subjects**	**Sex**	**Age**	**Educational level**	**Time post-onset**	**Type of aphasia**	**Noun naming**	**Verb naming**	**Noun compreh**	**Verb compreh**	**Token test**
B.C.	Female	63	8	3 year, 5 months	Non-fluent	5/30	2/28	40/40	20/20	16/36
F.S.	Female	71	5	1 year, 8 months	Non-fluent	3/30	3/28	40/40	20/20	22/36
P.C.	Male	65	9	1 year, 7 months	Non-fluent	6/30	6/28	40/40	20/20	9/36
P.F.	Male	44	13	7 years	Non-fluent	3/30	8/28	40/40	20/20	17/36
A.C	Male	64	13	4 years, 5 months	Non-fluent	4/30	2/28	40/40	20/20	19/36
N.M.	Female	65	13	3 years, 7 months	Non-fluent	5/30	3/28	40/40	20/20	18/36
P.M.	Male	52	13	1 year, 2 months	Non-fluent	6/30	4/28	40/40	20/20	12/36
R.L.	Male	61	11	4 years, 7 months	Non-fluent	3/30	4/28	40/40	20/20	10/36
R.F.	Male	53	13	7 months	Non-fluent	4/30	2/28	40/40	20/20	9/36
B.A.	Female	59	18	3 years, 3 months	Non fluent	6/30	3/28	40/40	20/20	16/36
P.E.	Male	68	18	1 year, 8 months	Non-fluent	5/30	4/28	40/40	20/20	11/36
M.A.	Male	50	18	4 years, 4 months	Non-fluent	8/30	6/28	40/40	20/20	13/36

Legend. Compreh, Comprehension.

For each language task, the number of correct responses are reported.

### Materials

Six short videoclips (15 min each) reproducing common everyday life situations were prepared for the therapy. Three of them were employed to elicit spontaneous speech during the treatment (T[reatment]-videoclips: two persons eating at the restaurant, people leaving at the station, a woman attending to household chores). The remaining three videoclips were presented to the participants only before and after the therapy to control for generalization effects (G[eneralization]-videoclips: a girl making a coffee at home, a woman shopping at the supermarket, the housekeepers cleaning inside a hotel).

### Procedure

Prior to the experiment, all six videoclips (T- and G-videoclips) were shown to the control group. Each participant was asked to freely describe each video accurately, with no interference from the examiner.

### Transcranial direct current stimulation (tDCS)

tDCS was applied using a battery driven Eldith (neuroConn GmbH) Programmable Direct Current Stimulator with a pair of surface-soaked sponge electrodes (5 × 7 cm). A constant current of 1 mA intensity was applied on the skin for 20 min. If applied according to safety guidelines, tDCS is considered to be a safe brain stimulation technique with minor adverse effects (Poreisz et al., 2007). Two different electrode stimulation positions were used: the F5 of the extended International 10–20 system for EEG electrode placement, which correspond best to the Broca's area (Nishitani et al., 2005; Naeser et al., 2010) and the CP5 of the extended International 10–20 system for EEG electrode placement, which has been found to correspond best to the Wernicke's area (Oliveri et al., 1999; Fiori et al., 2011). In both conditions the reference electrode was placed over the contralateral frontopolar cortex (Nitsche and Paulus, 2000; Sparing et al., 2008).

**Figure 2:**
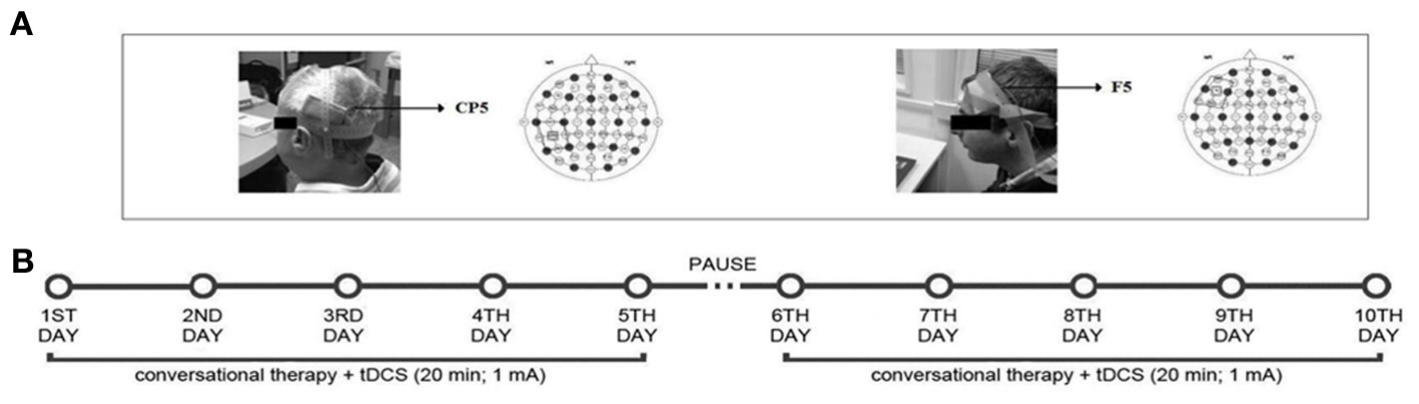
**Localization of the tDCS area (A) and overview of study design (B): one video-clip was used for the anodic Broca's stimulation, one for the anodic Wernicke's stimulation and a third one for the sham Broca's or Wernicke's condition.** Each condition was performed in 10 consecutive daily sessions over 3 months, with 14 days of intersession interval, while the subjects underwent the “Conversational Therapy” treatment.

The persons with aphasia underwent two different stimulation conditions: (1) anodic (F5-A) stimulation over the Broca's area; (2) anodic (CP5-A) stimulation over the Wernicke's area; a sham condition was also included (F5/CP5 S). The sham condition was performed exactly like anodic stimulation. To better simulate the two stimulation conditions in half of the participants the electrode was applied over the Broca's area, whereas in the remaining half over the Wernicke's area. In both conditions, the stimulator was turned off after 30 s. It has been shown that this procedure makes it possible to blind subjects as to the respective stimulation condition (Gandiga et al., 2006). Each stimulation condition was performed with concurrent speech therapy (the Conversational therapy approach). Although tDCS stimulation was delivered from the beginning of the therapy sessions up to 20 min, the language treatment lasted 2 h per day, in 10 consecutive daily sessions (Monday–Friday, weekend off, Monday–Friday). There was a 14-day intersession interval between each condition (see Figure 2).

During the language treatment, each T-videoclip was assigned to a different stimulation condition. The order of presentation of the T-videoclips and of stimulation conditions was randomized across subjects. The randomization procedure was delivered through allocation concealment. A clinician not involved in the rest of the study assigned each participant to the stimulation's condition. The random sequence was generated using sequentially numbered, opaque, sealed envelopes. Each language sample was tape-recorded and transcribed verbatim by a clinician. Both the person with aphasia and the clinician were blind with respect to the administration of tDCS. At the end of each condition, subjects were asked if they were aware of which condition (real or sham) they had been exposed to. None of the subjects was able to ascertain differences in intensity of sensation between the two conditions. To measure baseline performance, at the beginning of each experimental condition, all participants were asked to describe the T-videoclip without the therapist's help. The same was done at the end of each experimental condition.

### Language treatment

According to the Conversational Therapy approach, the main goal of the clinician is to set up a natural conversation with the person with aphasia in which both interlocutors participate using their available communicative resources. Both the aphasic and the therapist were left free to use any communicative means (e.g., gestures, drawings, orthographic or phonological cues) to exchange salient information about the videoclip. The therapist was instructed to accept all the information provided by the patient and tried to relate it to the topic of conversation in order to improve its content and informative level. The goal of the therapy was to make the person with aphasia as much informative as possible on a daily basis and to bring him/her to talk about the video without the therapist's support.

In order to measure generalization of treatment effects, at the beginning and at the end of each experimental condition, all participants were re-administered the language tests and asked to describe the three G-videoclips without the therapist's support. Each language sample was tape-recorded and transcribed verbatim by two independent transcribers. The transcriptions were then compared so to obtain highly-reliable discourse samples that we could segment and analyze. The scoring procedure was performed independently by two raters and then compared. Reproducibility of the scoring procedures resulted in substantial agreement among the coders. The few discrepancies were resolved through discussion.

### Follow-up

At 1 month after the end of each experimental condition, all subjects were again shown the corresponding T-video and asked to describe it without help. Also in this case, each language sample was tape-recorded and transcribed verbatim.

### Data analysis

Data were analyzed with SPSS 13.0 software. For the healthy and aphasic group, the mean number of C-Units, verbs and sentences produced for each T and G videoclip is reported in Table 2. Since during the treatment the videoclips were randomized across subjects and conditions, for the T-videoclips the data are reported only for the pre-and-post treatment sessions.

**Table 2:** **Mean number (± Standard Deviation) of C Units, Verbs, Sentences for the control and aphasic group collected in each T- and G-Videoclips**.

	**Control group**	**Aphasic group PRE-POST treatment**		**Control group**	**Aphasic group PRE/POST treatment**		**Control group**	**Aphasic group PRE/POST treatment**	
	**C-Units**	**C-Units**	**C-Units**	**Verbs**	**Verbs**	**Verbs**	**Sentences**	**Sentences**	**Sentences**
**T-VIDEOCLIPS**									
Eating at the restaurant	38 (±9)	7^**^ (±6)	14^*^/^**^ (±11)	45 (±10)	14^**^ (±9)	21^*^/^**^ (±13)	35 (±8)	6^**^ (±6)	14^*^/^**^ (±11)
At the station	35 (±9)	9^**^ (±6)	16^*^/^**^ (±8)	44 (±10)	24^**^ (±11)	31^*^/^**^ (±12)	31 (±9)	8^**^ (±5)	13^*^/^**^ (±7)
At home	40 (±9)	9^**^ (±5)	17^*^/^**^ (±10)	47 (±11)	18^**^ (±9)	27^*^/^**^ (±12)	37 (±8)	9/^**^ (±5)	16^*^/^**^ (±9)
**G-VIDEOCLIPS**									
Making a coffee									
**Broca**	15 (±5)	5 (±5)^**^	10 (±6)^*^/^**^	13 (±4)	9 (±8)	12 (±8)^*^	13 (±3)	4 (±4)^**^	5(±6)^**^
**Wernicke**		6(±5)^**^	6(±4)^**^		9(±6)	9(±6)		3(±2)^**^	3(±3)^**^
**Sham**		5(±5)^**^	5(±5)^**^		9(±7)	8(±7)		3(±3)^**^	3(±2)^**^
Shopping at the supermarket									
**Broca**	14 (±4)	2 (±2)^**^	6(±5)^*^/^**^	14 (±4)	5 (±4)^**^	6(±6)^**^	14 (±3)	2 (±2)^**^	6(±4)^*^/^**^
**Wernicke**		2(±3)^**^	3(±3)^**^		5(±4)^**^	5(±3)^**^		1(±1)^**^	2(±2)^**^
**Sham**		3(±3)^**^	2(±2)^**^		5(±5)^**^	5(±4)^**^		2(±2)^**^	1(±1)^**^
The housekeepers									
**Broca**	14 (±2)	3 (±3)^**^	4(±6)^**^	15 (±4)	7 (±6)^**^	9(±6)	12 (±4)	2 (±2)^**^	6(±5)^*^/^**^
**Wernicke**		4(±3)^**^	5(±3)^**^		8(±4)^**^	7(±5)^**^		3(±2)^**^	4(±3)^**^
**Sham**		3(±3)^**^	3(±3)^**^		7(±6)^**^	7(±5)^**^		3(±3)^**^	3(±2)^**^

Since during the treatment the videoclips were randomized across subjects and conditions, for the T-videoclips data are reported only for the pre-post treatment sessions (pre/post treatment sessions in the aphasic group all paired t-test

* p < 0.05; across groups

** p < 0.01).

Before and after each treatment session, the mean number (and standard deviation) of correct C-Units, verbs and sentences produced by each aphasic in the T-and-G videoclips was divided by the mean number collected in the healthy control group for the same linguistic variables and videoclips. The final result was converted into a mean percentage of correct responses and then analyzed.

In the aphasic group, two different analyses were run: the former focused on the results achieved before and after therapy using the videoclips as treatment materials (T-videoclips); the latter focused on the generalization effects obtained on the videoclips presented only before and after the therapy (G-videoclips). For each analysis, a 2 × 3 repeated-measures ANOVA (ANOVA_rm_) with two within-subject factors: *Time* [baseline (T1) vs. end of treatment (T10)] and *Condition* (anodic Broca's area vs. anodic Wernicke's area vs. Sham) was run separately for C-Units, verbs and sentences. The Interaction was explored by using the Scheffè post-hoc test.

In addition, to measure long-lasting beneficial effect of the treatment a 2 × 3 × 3 repeated-measures ANOVA (ANOVA_rm_) with three within-subject factors: *Time* [end of treatment (T10) vs. follow up (F1)], *Condition* (anodic Broca's area vs. anodic Wernicke's area vs. Sham) and *Category* (Verbs vs. C-Units vs. Sentences) was run. The Interaction was explored by using the Scheffè *post-hoc* test.

Finally, before and after each treatment session, the aphasic's responses to the different re-administration of the standardized language tests were analyzed. Since no significant differences were found in each language task (chi square tests, all *ps* = n.s.), the data were not further investigated.

## Results

### Treatment

#### C-units

**Figure 3:**
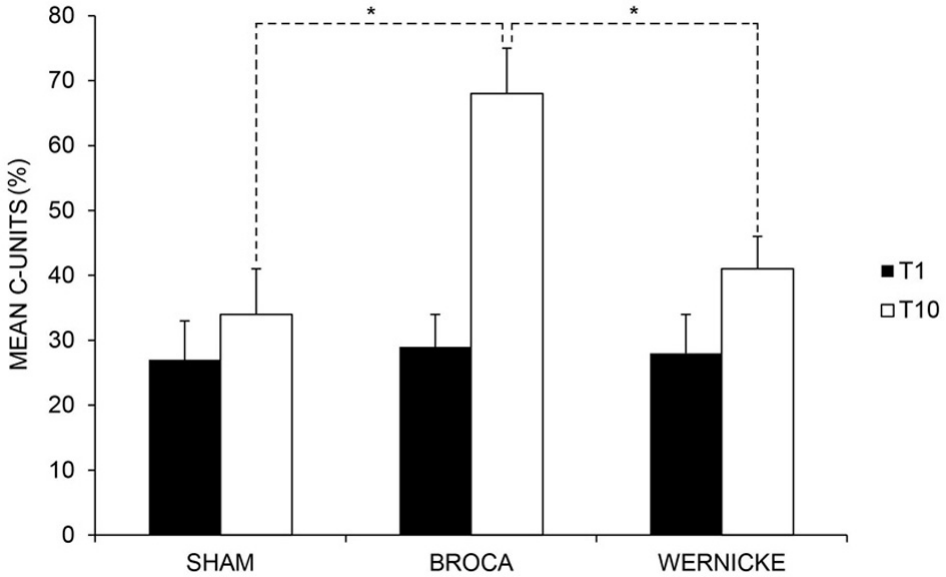
Mean percentage of correct “C-Units” at baseline (T1) and at the end of treatment (T10) for the left anodic Wernicke's, Broca's and sham conditions (^*^< 0.01), respectively. Error bars represent standard error of the mean.

The analysis showed a significant effect of *Time* [baseline (T1) vs. end of treatment (T10), *F*_(1, 11)_ = 51.50; *p* = 0.000] and of *Condition* [anodic Broca's area vs. anodic Wernicke's area vs. Sham, *F*_(2, 22)_ = 5.22; *p* = 0.014]. Subjects' performance significantly improved at the end of training with respect to baseline [mean = 48%, SEM = 6 (T10) vs. mean = 28%, SEM = 4 (T1) *p* = 0.000]. Moreover, the mean percentage of C-Units in the anodic Broca's condition was significantly greater than in the other two conditions (mean = 48%, SEM = 7 (anodic Broca's) vs. mean = 35%, SEM = 4 (anodic Wernicke's) vs. mean = 31%, SEM = 6 (Sham) *p* = 0.014). The interaction of *Time × Condition* [*F*_(2, 22)_ = 24.18; *p* = 0.000] was also significant. While no significant differences emerged in the mean percentage of C-Units between the three conditions at baseline (differences between Broca vs. Wernicke = 1%, *p* = 0.768; differences between Broca vs. Sham = 2%, *p* = 0.509; differences between Wernicke vs. Sham = 1%, *p* = 0.713), at the end of training, the mean percentage of C-Units was significantly greater in the anodic Broca's condition with respect to the other two conditions, which did not differ from each other (differences between Broca vs. Wernicke = 27%; *p* = 0.000; differences between Broca vs. Sham = 34%; *p* = 0.000; differences between Sham vs. Wernicke = −7%; *p* = 0. 064) (see Figure 3 and Table 3).

**Table 3:** **Mean percentage of correct C-Units, Verbs and Sentences (±SEM) for the 12 aphasic participants at the baseline (T1), at the end of treatment (T10) and at follow-up (1 month after the end of treatment) for the Broca's, Wernicke's and sham condition, respectively (SEM = error standard of the mean)**.

	**BROCA**	**WERNICKE**	**SHAM**	**Total mean**
**C-UNITS**				
T1	29 (±6)	28 (±4)	27 (±6)	28 (±4)
T10	68 (±9)	41 (±5)	34 (±7)	48 (±6)
FOLLOW UP	64 (±9)	43 (±6)	36 (±7)	48 (±6)
Total mean	53(± 8)	37 (± 5)	32 (±7)	
**VERBS**				
T1	28 (±5)	31 (±6)	27 (±6)	29 (±4)
T10	62 (±7)	40 (±5)	37 (±7)	47 (±3)
FOLLOW UP	60 (±6)	41 (±5)	38 (±6)	47 (±3)
Total mean	50 (±5)	37 (±5)	34 (±6)	
**SENTENCES**				
T1	32 (±7)	28 (±4)	28 (±7)	30 (±5)
T10	67 (±9)	40 (±6)	36 (±7)	48 (±6)
FOLLOW UP	65 (±9)	40 (±6)	35 (±7)	47 (±6)
Total mean	55 (±8)	36 (±5)	33 (±7)	

#### Verbs

**Figure 4:**
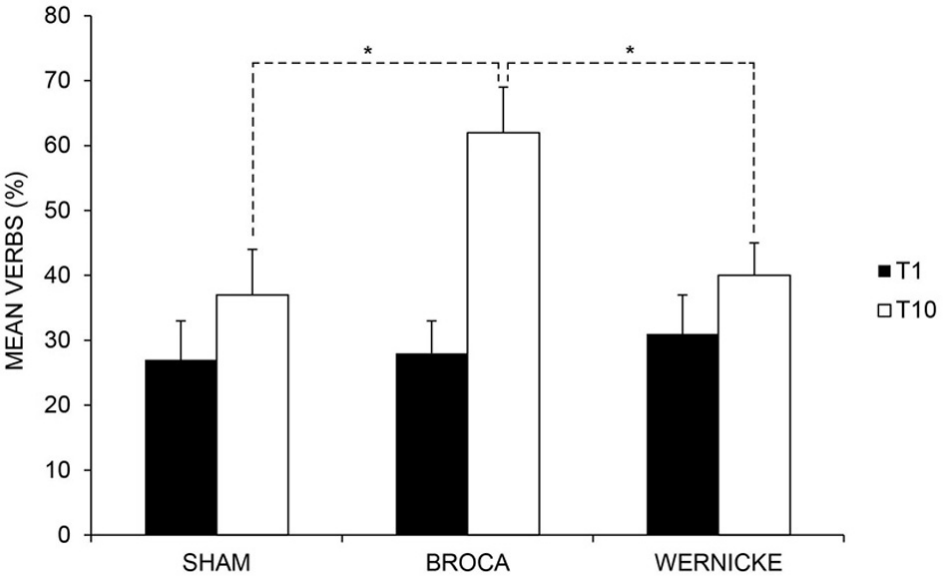
Mean percentage of correct verbs at baseline (T1) and at the end of treatment (T10) for the left anodic Wernicke's, Broca's and sham conditions (^*^< 0.01), respectively. Error bars represent standard error of the mean.

The analysis showed a significant effect of *Time* [baseline (T1) vs. end of treatment (T10), *F*_(1, 11)_ = 34.53; *p* = 0.000] but not of *Condition* [anodic Broca's area vs. anodic Wernicke's area vs. Sham, *F*_(2, 22)_ = 1.54; *p* = 0.235]. Subjects' performance significantly improved at the end of training with respect to baseline [mean = 47%, SEM = 3 (T10) vs. mean = 29%, SEM = 4 (T1) *p* = 0.000]. The interaction of *T*ime × Condition [*F*_(2, 22)_ = 7.38; *p* = 0.004] was also significant. While no significant differences emerged in the mean percentage of verbs between the three conditions at baseline (differences between Broca vs. Wernicke = −3%, *p* = 0.588; differences between Broca vs. Sham = 1%, *p* = 0.950; differences between Wernicke vs. Sham = −4%, *p* = 0. 546), at the end of training, the mean percentage of verbs was significantly greater in the anodic Broca's condition with respect to the other two conditions, which did not differ from each other (differences between Broca vs. Wernicke = 22%; *p* = 0.000; differences between Broca vs. Sham = 25%; *p* = 0.000; differences between Sham vs. Wernicke = −3%; *p* = 0. 642) (see Figure 4 and Table 3).

#### Sentences

**Figure 5:**
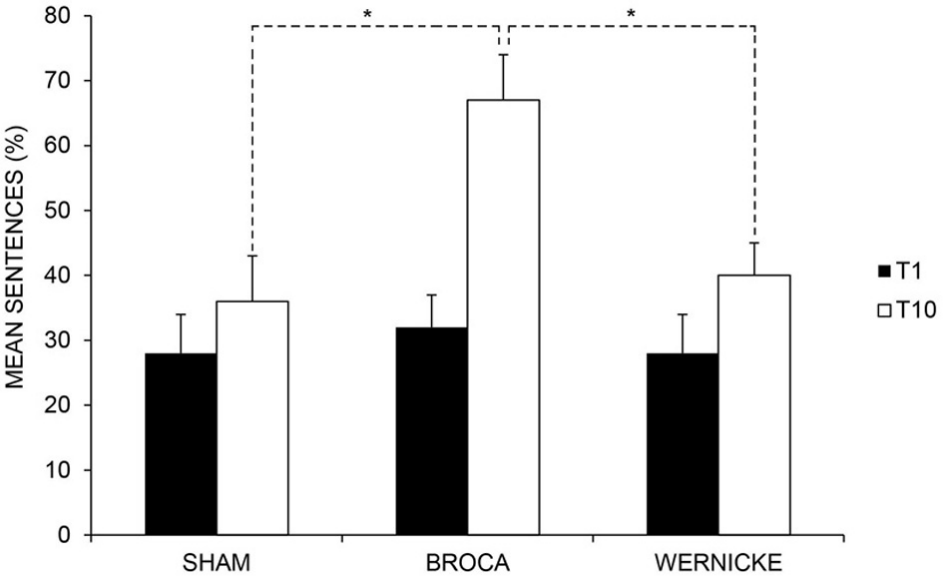
Mean percentage of correct sentences at baseline (T1) and at the end of treatment (T10) for the left anodic Wernicke's, Broca's and sham conditions (^*^< 0.01), respectively. Error bars represent standard error of the mean.

The analysis showed a significant effect of *Time* [baseline (T1) vs. end of treatment (T10), *F*_(1, 11)_ = 44.77; *p* = 0.000] and of *Condition* [anodic Broca's area vs. anodic Wernicke's area vs. Sham, *F*_(2, 22)_ = 6.29; *p* = 0.007]. Subjects' performance significantly improved at the end of training with respect to baseline [mean = 48%, SEM = 6 (T10) vs. mean = 30%, SEM = 5 (T1) *p* = 0.000]. Moreover, the mean percentage of sentences in the anodic Broca's condition was significantly greater than in the other two conditions (mean = 50%, SEM = 8 (anodic Broca's) vs. mean = 34%, SEM = 5 (anodic Wernicke's) vs. mean = 32%, SEM = 6 (Sham) *p* = 0.007). The interaction of *Time × Condition* [*F*_(2, 22)_ = 76.62; *p* = 0.000] was also significant. While no significant differences emerged in the mean percentage of sentences between the three conditions at baseline (differences between Broca vs. Wernicke = 4%, *p* = 0.238; differences between Broca vs. Sham = 4 %, *p* = 0.275; differences between Wernicke vs. Sham = 0%, *p* = 0.927), at the end of training, the mean percentage of sentences was significantly greater in the anodic Broca's condition with respect to the other two conditions, which did not differ from each other (differences between Broca vs. Wernicke = 27%; *p* = 0.000; differences between Broca vs. Sham = 31%; *p* = 0.000; differences between Sham vs. Wernicke = −4%; *p* = 0. 190) (see Figure 5 and Table 3).

### Generalization of the treatment

**Figure 6:**
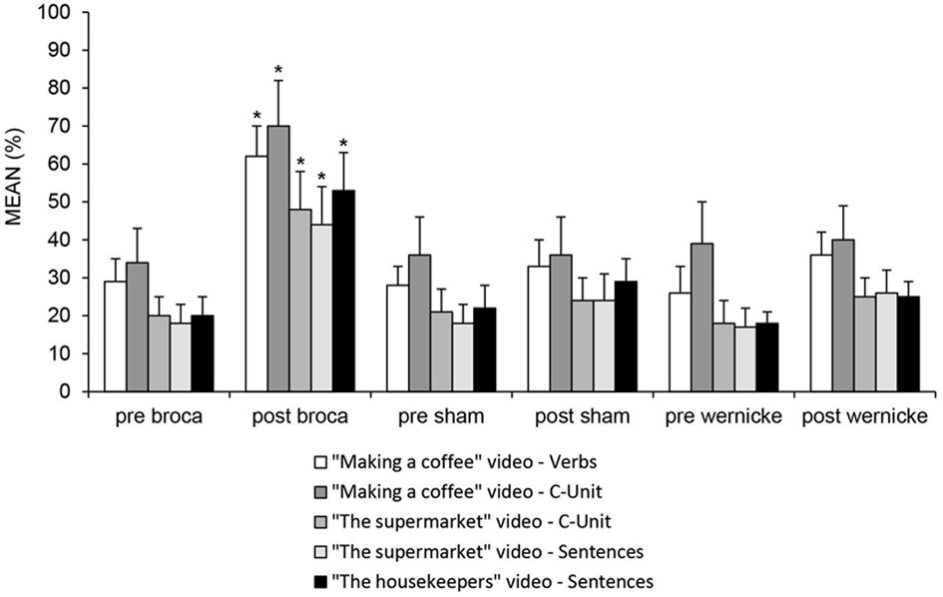
Mean percentage of correct “C-Units”, verbs and sentences during the presentation of the three videos (“Making a coffee”, “The Supermarket” and “The Housekeepers”) presented only at baseline and at the end of treatment in the pre-and post-anodic Broca's, sham and Wernicke's conditions (^*^< 0.01), respectively.

For the “Making a Coffee” video, we found a greater improvement in the Broca's condition with respect to the other two conditions in the mean number of verbs (differences between Broca vs. Wernicke = 26%; *p* = 0.000; differences between Broca vs. Sham = 29%; *p* = 0.000; differences between Sham vs. Wernicke = −3%; *p* = 0.604) and C-Units (differences between Broca vs. Wernicke = 30%; *p* = 0.000; differences between Broca vs. Sham = 34%; *p* = 0.000; differences between Sham vs. Wernicke = −4%; *p* = 0.559). An improvement in C-Units was also found for the “The supermarket” video, which was significantly greater in the Broca's condition with respect to the other two conditions (differences between Broca vs. Wernicke = 23%; *p* = 0.000; differences between Broca vs. Sham = 24%; *p* = 0.000; differences between Sham vs. Wernicke = −1%; *p* = 0.829). For the same video and again for the Broca's condition, we also observed a significant greater change in the mean number of correct sentences with respect to the other two conditions (differences between Broca vs. Wernicke = 18%; *p* = 0.000; differences between Broca vs. Sham = 20%; *p* = 0.002; differences between Sham vs. Wernicke = −2%; *p* = 0.705). This last result significantly changed in the same condition also for “The housekeepers” video (differences between Broca vs. Wernicke = 28%; *p* = 0.000; differences between Broca vs. Sham = 24%; *p* = 0.000; differences between Sham vs. Wernicke = 4%; *p* = 0.455) (see Figure 6).

The relationship between C-Units, Verbs and Sentences after stimulation of Broca's area was further investigated using Pearson product-moment correlation coefficient. Preliminary analyses were performed to ensure no violation of the assumptions of normality, linearity, and homoscedasticity. There was a strong positive correlation between C-Units and Verbs [*r* = 0.75; *p* = 0.005] and between verbs and sentences [*r* = 0.75; *p* = 0.005]. This suggests that the stimulation of Broca's area increased the production of informative units and that such increase boosted the production of verbs. Furthermore, the increased ability to have access to the morphosyntactic information contained in these verbs allowed them to produce more accurate sentences.

### Follow-up

**Figure 7:**
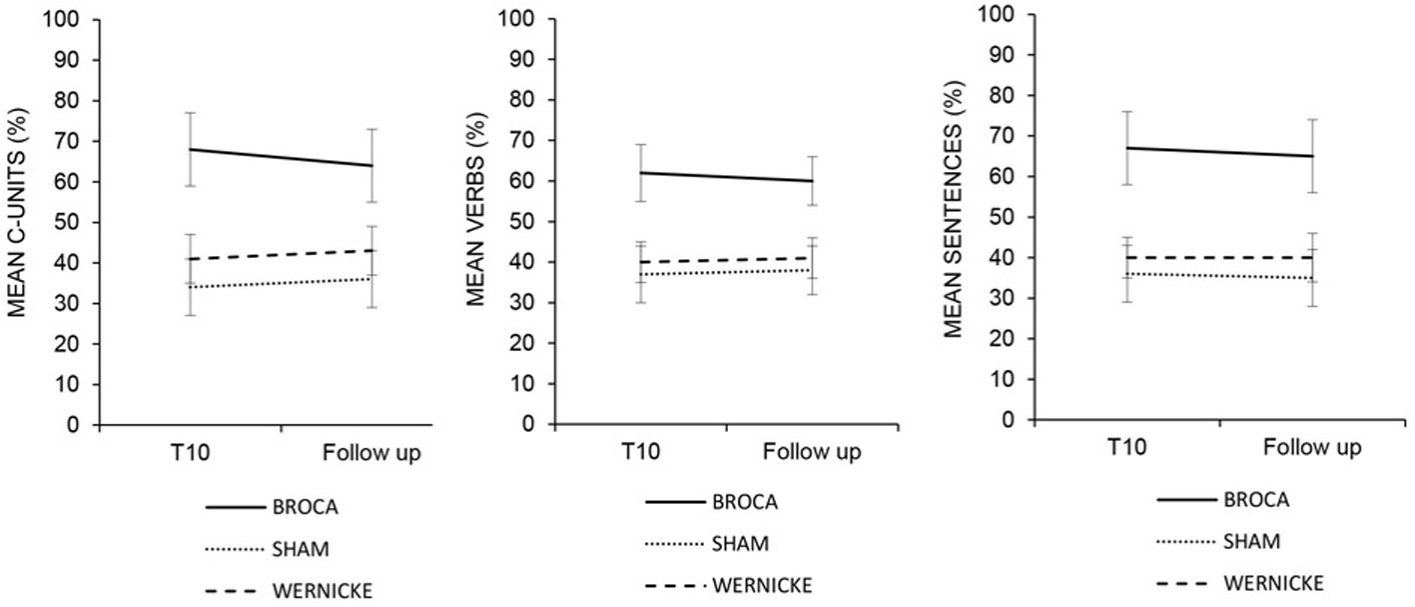
**Mean percentage of correct C-Units, Verbs and Sentences at the end of treatment (T10) and at the follow-up for the left anodic Wernicke's, Broca's and sham condition.** Error bars represent standard error of the mean.

Overall, the analysis showed a significant effect of *Condition* [Broca's vs. Wernicke's vs. Sham conditions, *F*_(2, 22)_ = 11.43; *p* = 0.000] indicating a greater improvement for C-Units, Verbs and Sentences production in the Broca's condition with respect to the other two conditions [Broca's (mean = 64%, SEM = 7) vs. Wernicke's (mean = 41%, SEM = 5) vs. Sham condition (mean = 36%, SEM = 6) *p* = 0.000]. Neither the *Time* [*F*_(1, 11)_ = 0.21; *p* = 0.654] nor the *Category* effect [*F*_(2, 22)_ = 0.04; *p* = 0.963] were significant suggesting a persistence of the results obtained at the end of treatment after 1 month for all categories (see Table 3 and Figure 7).

## Discussion

The aim of this study was to determine if tDCS delivered over the Broca's area coupled with an intensive treatment based on Conversational therapy improves informative speech in persons with chronic non-fluent aphasia. To keep high ecological validity and, therefore, to analyze communication in natural contexts, our materials included six videoclips depicting common everyday situations.

Overall, three major findings will be discussed: (1) after Broca's stimulation, the ability of the persons with aphasia to produce informative speech showed the greatest improvement as they produced descriptions with more C-Units; (2) changes in informativeness during therapy corresponded to relevant changes in the production of verb that, in turn, boosted the participants' ability to use relevant morphosyntactic information and increased the number of sentences produced; (3) significant changes after therapy persisted after 1 month and were observed not only on the videoclips used during the treatment but also in the three videoclips presented to the participants only at the beginning and at the end of the therapy sessions, while no changes were found on the standard aphasia assessment.

The production of informative messages is an effortful endeavor that relies on the interaction between microlinguistic (i.e., lexical and grammatical) and macrolinguistic (i.e., pragmatic and discourse) levels of processing. The goal of the therapy used in the present study was to encourage the use of informative speech even if not always formally correct. Therefore, the approach was mainly focused onto the pragmatic aspects of language. Indeed, patients were required to select the lexical representations that were appropriate to the given context and to organize them within a communicative interaction. To date, although the neural correlates of microlinguistic processing have been extensively studied (Vigneau et al., 2006), the investigation of the ability to organize the macrolinguistic aspects of message production have been much less explored. Recent neuropsychological, neuroimaging and fMRI studies have suggested that Broca's area and the adjacent portion of the left inferior frontal cortex may play a major role for the top-down controlled selection and/or retrieval of contextually adequate words from the mental lexicon (Loban, 1966; Wagner et al., 2001; Hagoort, 2005; Koechlin and Jubault, 2006; Lau et al., 2008; Kim et al., 2011; Whitney et al., 2011; Marini and Urgesi, 2012; Schuhmann et al., 2012). Once retrieved, this lexical information is unified into an overall representations that spans multi-word utterances (Indefrey and Cutler, 2005). As a result, by faciliting the process of lexical selection and retrieval, the stimulation of this region likely elicits the integration of word meanings into an unfolding discourse representation of the context (Hagoort, 2005). Our results are coherent with this interpretation. Indeed, after Broca's stimulation, the patients who were initially unable to communicate verbally could sustain a conversation and produce more content units (i.e., informative chunks). Recently, Marini and Urgesi (2012) reached similar conclusions. In their study, rTMS applied over the dorsal portion of the anterior left, but not right, inferior frontal gyrus (LIFG) reduced the levels of lexical informativeness of narratives produced by a group of healthy individuals. In the authors' interpretation, since the LIFG is involved in the selection of specific lexical concepts, the inhibition of this area through rTMS hampered this process forcing the speakers to change the flow of discourse so to occasionally produce utterances conceptually incongruent with the story.

However, since verbs carry critical meaning in the communicative process, after the treatment we also found a significant increase in verb production. This result is in line with recent reports which showed that excitatory stimulation applied over the left Broca's area or over the surrounding frontal region (left dorsolateral frontal cortex, LDLFC), together with simultaneous intensive language training, led to the greatest amount of verb naming improvement (Cotelli et al., 2012; Marangolo et al., 2013).

It is widely acknowledged that verb representation constrains the assignment of retrieved lexical items to positions within the syntactic frame, and, therefore, plays a crucial role in the structural formulation of sentences (Zingeser and Berndt, 1990; Wambaugh et al., 2002). Accordingly, the speech samples of our participants after the treatment included more grammatically correct sentences. Again, this improvement was greatest after the anodic stimulation over Broca's area. This result is in agreement with recent findings from fMRI studies on spontaneous production in healthy participants, which showed a significant association between syntactic skills and activation of the left inferior frontal gyrus (Grande et al., 2012; see also Menenti et al., 2012).

Taken together, these results suggest that Broca's area is a cortical epicenter subserving the selection of contextually appropriate semantic representations. This conceptual information triggers the generation of appropriate propositions that must be organized at the macrolinguistic level by means of coherent links and increases discourse informativeness.

One important finding of our study was that the enhanced recovery in the participants' ability to communicate, as measured in terms of C-Units, Verbs and Sentences, still persisted at 1 month after the end of treatment. Furthermore, this recovery was generalized also to other contexts which were presented to the participants only before and after each treatment condition.

It has been suggested that long-lasting functional changes in the cortex as the result of electrical stimulation are the consequence of modulation of the strength of synaptic connections (i.e., synaptic plasticity, Nitsche and Paulus, 2000). In our study, the choice to stimulate the left-language hemisphere regions was related to previous results indicating that the stimulation of these sites in persons with chronic aphasia might enhance functional improvement inducing the reactivation of left-hemispheric perilesional structures (Baker et al., 2010; Fiori et al., 2011; Fridriksson et al., 2011; Marangolo et al., 2011, 2013). These results agree with the hypothesis that, in individuals with chronic aphasia, language recovery mostly involved the left unaffected cortical areas (Saur et al., 2006; Warburton et al., 1999; Winhuisen et al., 2007). Although it is often assumed that the right homologue of Broca's area takes over the function of the left if it is infarcted, the evidence for this is slender. Recent studies have stressed the importance of left Broca's area or adjacent tissue in the natural recovery from post-stroke aphasia (Saur et al., 2006, 2008) and there is some evidence that the right homologue of Broca's area inhibits recovery (Naeser et al., 2005, 2010, 2011).

In our aphasic group, the lesion mapping analysis over the two stimulated areas allowed us to exclude that the better recovery observed after Broca's stimulation was due to a greater sparing of this region to cerebral damage compared to Wernicke's area. Indeed, the same percentage of damage was present into the two stimulated areas (45%) (see Figure 1) However, in those subjects in which Broca's area was partly or completely damaged, it might be speculated that tDCS has influenced the activity of the brain centers close to the stimulated site producing a rearrangement of synaptic efficiency within the underlying network which in turn led to an improvement of the cognitive language ability (Miniussi et al., 2008; Cotelli et al., 2011).

One final comment regards the fact that traditional standardized language tests failed to capture the significant post-therapy effect that was evident during the descriptions of the videoclips. Likely, this discrepancy might be due to the specific nature of these different tasks. Indeed, while traditional language tests require to produce words under the administration of static pictures, the dynamic therapeutic setting devised for this study employed videoclips representing highly realistic contexts that exerted a positive influence on lexical retrieval. Overall, these considerations are in line with the hypothesis that a multi-level approach to language analysis is more adequate than standardized language tests to quantify communicative improvement in persons with aphasia (Larfeuil and Le Dorze, 1997; Marini et al., 2007, 2011; Andreetta et al., 2012).

In conclusion, we believe that our data clearly show that the recovery of language in persons with aphasia can be successfully enhanced in different linguistic domains by coupling specific treatment approaches with relatively simple stimulation procedures such as tDCS.

## Author contributions

Conceived and designed the experiments: Paola Marangolo, Valentina Fiori. Performed the experiments: Valentina Fiori, Carmelina Razzano. Analyzed the data: Valentina Fiori, Maria Antonietta Calpagnanot. Analyzed neuroimaging data Serena Campana. Wrote the paper: Paola Marangolo. Editing and critical revision of the manuscript: Andrea Marini, Carlo Caltagirone.

### Conflict of interest statement

The authors declare that the research was conducted in the absence of any commercial or financial relationships that could be construed as a potential conflict of interest.
